# ChemoDOTS: a web server to design chemistry-driven focused libraries

**DOI:** 10.1093/nar/gkae326

**Published:** 2024-04-30

**Authors:** Laurent Hoffer, Guillaume Charifi-Hoareau, Sarah Barelier, Stéphane Betzi, Thomas Miller, Xavier Morelli, Philippe Roche

**Affiliations:** CRCM, CNRS, Inserm, Institut Paoli-Calmettes, Aix-Marseille Univ, Marseille 13273, France; CRCM, CNRS, Inserm, Institut Paoli-Calmettes, Aix-Marseille Univ, Marseille 13273, France; CRCM, CNRS, Inserm, Institut Paoli-Calmettes, Aix-Marseille Univ, Marseille 13273, France; CRCM, CNRS, Inserm, Institut Paoli-Calmettes, Aix-Marseille Univ, Marseille 13273, France; CRCM, CNRS, Inserm, Institut Paoli-Calmettes, Aix-Marseille Univ, Marseille 13273, France; CRCM, CNRS, Inserm, Institut Paoli-Calmettes, Aix-Marseille Univ, Marseille 13273, France; CRCM, CNRS, Inserm, Institut Paoli-Calmettes, Aix-Marseille Univ, Marseille 13273, France

## Abstract

In drug discovery, the successful optimization of an initial hit compound into a lead molecule requires multiple cycles of chemical modification. Consequently, there is a need to efficiently generate synthesizable chemical libraries to navigate the chemical space surrounding the primary hit. To address this need, we introduce ChemoDOTS, an easy-to-use web server for hit-to-lead chemical optimization freely available at https://chemodots.marseille.inserm.fr/. With this tool, users enter an activated form of the initial hit molecule then choose from automatically detected reactive functions. The server proposes compatible chemical transformations via an ensemble of encoded chemical reactions widely used in the pharmaceutical industry during hit-to-lead optimization. After selection of the desired reactions, all compatible chemical building blocks are automatically coupled to the initial hit to generate a raw chemical library. Post-processing filters can be applied to extract a subset of compounds with specific physicochemical properties. Finally, explicit stereoisomers and tautomers are computed, and a 3D conformer is generated for each molecule. The resulting virtual library is compatible with most docking software for virtual screening campaigns. ChemoDOTS rapidly generates synthetically feasible, hit-focused, large, diverse chemical libraries with finely-tuned physicochemical properties via a user-friendly interface providing a powerful resource for researchers engaged in hit-to-lead optimization.

## Graphical Abstract

**Graphical Abstract:**
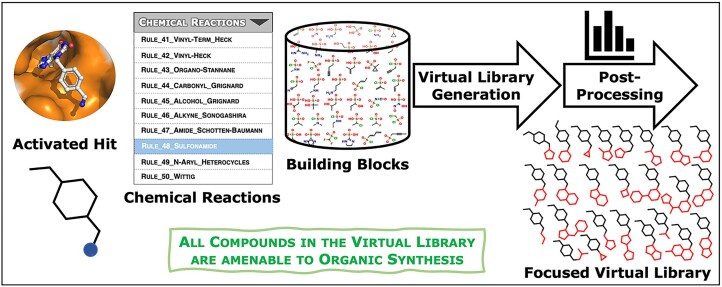

## Introduction

While the identification and validation of hit compounds is a nontrivial first step in the drug discovery process, these compounds normally require subsequent optimization to improve their efficacy, selectivity, and drug-like properties to become chemical probes or therapeutic candidates. During this optimization phase, vast chemical spaces are explored to find analogs or derivatives of the initial hit compounds with improved potency, selectivity, and better pharmacological properties. However, this hit-to-lead (H2L) optimization phase is usually a major bottleneck in drug discovery campaigns (1). Computational methods are increasingly being integrated into the H2L optimization process to aid in predicting compound properties, optimizing chemical structures, and prioritizing analogs for synthesis (1–5). One widely established approach to optimize validated hits is known as ‘growing’. This involves expanding chemical structures to introduce new interactions with the target, in order to improve potency and selectivity (6,7). However, a notable drawback emerges because of this strategy where the molecular weight of the compounds increases, outpacing gains in target affinity or activity, and leading to a phenomenon known as ‘molecular obesity’ (8,9). Therefore, it is crucial to initiate the H2L optimization phase with so-called ‘ligand-efficient’ molecules exhibiting a low molecular weight and high activity or potency. Such compounds can be obtained through the deconstruction or structural simplification of larger validated hit molecules (10,11) or starting with lower molecular weight compounds. Therefore, fragment-based drug discovery (FBDD) appears as one of the most relevant approaches to identify initial hits (12–16). Fragments are low molecular weight chemical compounds defined by the rule of three (17,18). One of the key advantages of fragment libraries, compared to standard chemical libraries used in high-throughput screenings, lies in the fact that a small number of fragments can effectively represent a vast chemical space (19,20). In addition, fragments also exhibit high hit rates compared to larger compounds. Because of the small size and low complexity of fragment molecules, FBDD is often combined with structure-based approaches to optimize the initial hits and increase their efficiency and specificity (21,22). One important aspect of the H2L optimization strategy is considering the synthetic feasibility of the designed compounds. Indeed, each iteration of the growing strategy, aimed at exploring the chemical space around the initial hit, involves synthesizing the designed analogs and testing them experimentally. Achieving this is typically performed through two main strategies. The synthetic feasibility can be estimated using machine learning approaches and expressed through a retrosynthetic score (23). Alternatively, carefully selected chemical reactions can be employed to virtually design potentially optimized compounds, as demonstrated by the curated collection proposed by Hartenfeller *et al.* (24). In this collection, 58 medicinal chemistry-relevant chemical reactions commonly used during the H2L optimization stage in the pharmaceutical industry have been encoded in the machine-readable SMARTS format. These reactions have been implemented in several approaches including DOGS (25), CROSS (26), PINGUI/SCUBIDOO (27,28), AutoCouple (29), NAOMINEXT (30), CHIPMUNK (31), SwissDrugDesign (32), AutoGrow4 (33), OpenChemLib (34) and eXplore (35). We also implemented this collection of reactions into our integrated drug design strategy called diversity-oriented target-focused synthesis (DOTS, (36)). The DOTS approach combines the design of focused chemical libraries and virtual sampling to prioritize the best potential optimizations. Briefly, after hit identification and characterization of its binding mode using structural biophysics method, such as X-ray crystallography, a virtual library is generated. This is achieved by combining an activated core analog of a hit fragment, with a collection of functionalized building blocks (BBs) using the chemical reactions previously defined by Hartenfeller *et al.* (24). Post-processing stages are then applied to extract a diverse subset of representative compounds that possess reasonable physicochemical properties and are devoid of any undesirable chemical functions. The final library undergoes virtual screening with the S4MPLE tool (37,38) to identify the best putative optimizations, aiming to create additional favorable contacts while maintaining the original binding mode. The DOTS approach has been successfully applied to various targets including the zika virus NS5 protein (39), syntenin PDZ domain protein (40–42), bromodomain-containing protein 4, BRD4 (43) and, more recently, a nucleotide kinase, dCK (44).

As demonstrated above, the ensemble of chemical reactions defined by Hartenfeller *et al.* is widely used in the chemoinformatics community. However, there is currently no easy-to-use freely accessible web server able to efficiently generate large ready-to-dock virtual libraries using these chemical reactions. In response to this gap, we introduce the ChemoDOTS web server designed to facilitate the generation of focused virtual libraries starting from a user-defined activated fragment. The user first proceeds by uploading or drawing a hit fragment in a sketcher. The web server then automatically identifies the chemical functions that permit the virtual coupling of BBs on the starting molecule. The user then selects one of the identified chemical functions and chooses from a list of compatible chemical reactions. Subsequently, a raw chemical library corresponding to the given reactions and compatible BBs is generated. At this stage, the user is given the option to apply a detailed set of post-processing filters to extract drug-like compounds with specific physicochemical properties. The final stage involves the computation of explicit stereoisomers and tautomers and the generation of a 3D conformer for each compound. A resulting virtual library compatible with most docking software is supplied as mol2 files with atom types and partial charges. The web server is freely accessible to all users, including commercial users, at the following address http://chemodots.marseille.inserm.fr/.

## Materials and methods

### Web server architecture

The web server is composed of two parts: the frontend, which handles user interactions, and the backend, which performs the computations. The frontend is a single-page application (SPA) designed with the React framework and embedding the MarvinJS sketcher version 22.11.1 from Chemaxon (https://chemaxon.com/). The backend server is written in Rust to speed up response time. It runs under a virtualized (KVM) Linux instance with a 4 cores/8 hyperthreads Intel CPU, 32 GB of RAM and 90 GB of storage. It makes use of a PostgreSQL database to store all the internal data (e.g. chemical functions, encoded chemical reactions, building blocks) and user-submitted data.

The raw compound references were stored separately from the building blocks to facilitate the treatment of duplicate molecules within or across providers while maintaining traceability. Chemical reactions, building blocks, and starting fragments were stored in their source/human readable format (SMARTS for reactions, SMILES for building blocks and fragments) to allow extensibility and direct compatibility with external tools. Additionally, they were stored in the RDkit pickle binary format which does not require sanitization and reduces loading time. Each operation was heavily parallelized using a thread pool across all available CPU cores to guarantee maximum performance. This was achieved through Rust's fearless concurrency mechanisms and the Rayon crate for parallel iterators. Database indexes were carefully defined to avoid computational bottlenecks without increasing the database size and reindexing workload for the database management system. To prevent unnecessary calculations, the list of building blocks available for each reaction was precomputed and stored within the database. The combined use of these optimizations allows fast chemical library generation producing at least 50 000 compounds per minute per reaction.

### Generation of raw chemical library

All the chemoinformatics operations are performed using the RDKit framework version 2023.09.5 (https://www.rdkit.org/) unless otherwise stated.

#### Preparation of building blocks

The list of 626 026 commercially available building blocks as of 2024-02 was retrieved from MolPort (https://www.molport.com/) as an SDF file. Then, a standardization pipeline was applied to discard undesirable compounds. First, for each compound, the largest organic fragment was selected discarding salts. The compounds containing only organic atoms (H, C, N, O, P, S), halogens (F, Cl, Br, I) or other atoms relevant in medicinal chemistry (B, Sn) were kept while explicit isotopes were filtered out. A series of filters were then applied to only retrieve BBs with <2 unspecified stereocenters, less than 17 rotatable bonds to limit flexibility, less than 4 rings and between 5 and 24 heavy atoms to restrict the molecules size to an acceptable range. Finally, the compound charges were neutralized. The standardized building blocks were converted into canonical SMILES allowing duplicates to be identified and grouped together with the same internal reference. The resulting chemical structures were serialized using the RDKit pickle format for faster loading from database storage. In the end, 501 542 unique building blocks from MolPort were kept. The same pipeline was applied for the 1 254 866 commercially available building blocks as of 2024-02-05 from the Enamine comprehensive catalog (https://enamine.net/building-blocks/building-blocks-catalog), resulting in the selection of 988 112 unique building blocks.

#### Detection of reactive functions

Reactive functions are detected by matching each building block against the chemical reactions. A substructure match is performed between an expected reactant template and a building block so that all the reacting building blocks for a given reaction are pre-identified and stored inside the database.

#### List of chemical reactions

The list of 58 SMART-encoded reactions was taken from the publication of Hartenfeller *et al.* (24). Several in-house reactions (numbered 61–70) used in internal projects have been added to overcome some exceptions in the original list of reactions. The complete list of reactions and their corresponding SMARTS are provided in Supplementary Tables S3–S5.

#### Detection of compatible chemical reactions

To identify reactions compatible with a selected chemical function on a provided fragment, the reactant templates from the reaction SMARTS are matched against the fragment to identify the reacting atoms. Only reactions for which one template shares at least one common atom with the function are kept.

#### Generation of raw chemical library

All fragment atoms not involved in the reaction are masked to prevent reactions with unwanted functions and to speed up the process. Next, the reaction products are generated. Building blocks leading to more than one distinct product are discarded. The products are then grouped by canonical SMILES to identify duplicates (if any) and the 2D coordinates are generated. At this stage, a set of molecular descriptors are computed. These descriptors are the fraction of sp^3^ carbons over the total carbon count (Fsp^3^), the number of hydrogen bond donors (HBD), the number of hydrogen bond acceptors (HBA), the logarithm of the predicted partition coefficient between octanol and water (cLogP), the average molecular weight (MW) and the topological polar surface area (TPSA). The products in SMILES/pickle formats and their descriptors are stored inside the database. Finally, the building blocks and products are exported to downloadable SDF and SMILES files.

#### Statistics of raw chemical library

At each step of the library generation, counters are implemented for individual reactions to report statistics, including the number of reacted building blocks, the number of raw products, the number of duplicates and the number of final unique products. These reaction-specific counters are merged to derive overall statistics for the generated chemical library.

### Post-processing stage

#### Filtering using physico-chemical descriptors

Histograms showing the frequency distribution of compounds are produced using molecular descriptors computed during the raw library generation. For discrete descriptors (HBA, HBD), a single bin is assigned for each unique value within the range of the descriptor. In the case of continuous descriptors (Fsp^3^, cLogP, MW, TPSA) 15 bins are specified. Then, the number of compounds matching a user-defined interval is computed in real time for each descriptor. This involves querying the database using descriptors indexes to retrieve the relevant information.

#### Generation of 2D library

The available products are collected from the database, then filtered using the intervals defined during the post-processing stage, sorted by molecular weight, and exported to downloadable SMILES and SDF files.

#### Generation of 3D library

The filtered products are fetched from the database and the major microspecies are computed at pH 7 using a simplified pKa model derived from OpenBabel (45). The protonation states predicted by OpenBabel are not always reliable. We intend to use other freely available tools in future versions. We are currently investigating different approaches including SPORES (46) and AI-based tools (47–49). An embedding step is performed to quickly generate a 3D conformation of the molecule with a correct geometry using ETKDG from RDKit (50). The validity of the generated conformations can be verified using PoseBusters, a Python package that performs standard quality checks using RDKit (51). A 3D SDF file is then exported. In addition, the atom types are inferred according to Corina, and Gasteiger partial charges are computed to generate a ready-to-dock mol2 file. It is worth noting that, to reduce computational times, the energy of the generated conformers is currently not minimized using any forcefield. However, we are actively exploring the addition of an optional step for efficient energy minimization to optimize the 3D structure of the generated molecules.

## Program description

### Design of the raw focused virtual library

**Figure 1.**
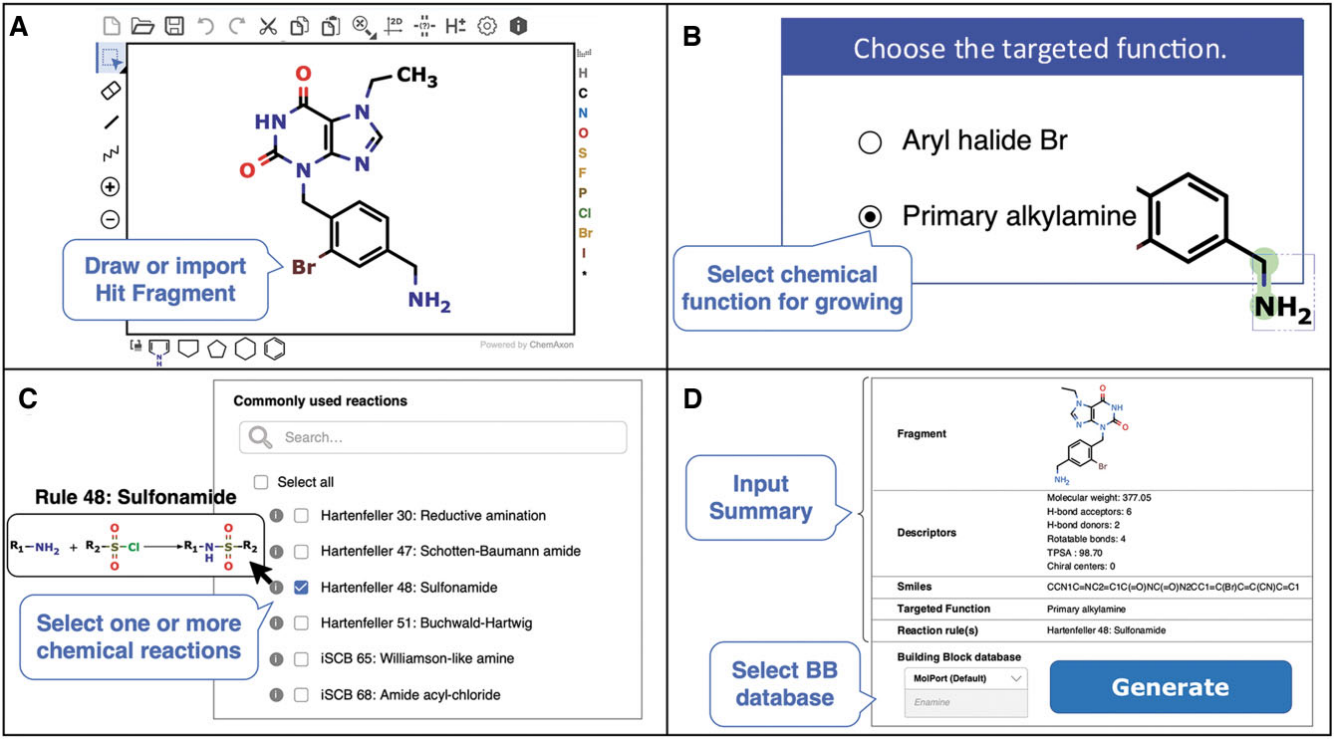
Overview of the generation of virtual libraries workflow. (**A**) First, the user is prompted to draw or upload the structure of the initial hit fragment into the Marvin JS sketcher. Standard formats such as SMILES, SDF, or PDB are recognized. (**B**) In the second step, all chemical functions compatible with the growing mode are automatically detected. The user should select the desired function for growing. (**C**) All chemical reactions compatible with the chosen chemical function are shown. They are subdivided into three categories depending on their relevance. A text box is provided to search for specific chemical reactions using rule ID numbers or keywords. The different chemical reactions can be highlighted by hovering on the information icons. The user should select at least one chemical reaction before proceeding to the next step. For example, they may choose to perform sulfonamidation, such as the chemical reaction between a primary alkylamine and sulfonyl chlorides (rule 48 defined by Hartenfeller). (**D**) A summary of the selected input is provided along with key molecular descriptors commonly used in early drug discovery. The user should select the building block database that will be used to generate the focused chemical libraries. The raw libraries are generated in seconds to minutes depending on the number of reactions selected and the number of compatible building blocks. In the given example, all selected building blocks contain a sulfonyl chloride function.

The overall workflow for designing the raw virtual library can be divided into 4 main steps summarized in Figure 1. First, the user must draw or upload the structure of the initial fragment (Figure 1A) using the provided Marvin JS sketcher from ChemAxon (https://chemaxon.com/). The SMILES code of the user-defined molecule is automatically generated. It is important to note that this reference fragment should contain at least one chemical function compatible with the fragment growing approach. A list of compatible functions is provided via the ‘Reactions’ link on the website, as well as in Supplementary Table S1 and S2 and in Figure S1. Two examples of molecules are provided to assist the user and can be directly imported into the sketcher (Figure 1A). In the next step, chemical functions present in the provided molecule are automatically detected and highlighted in the sketcher upon selection (Figure 1B). Users are then prompted to choose the chemical function for the fragment growing approach. For each chemical function, a list of compatible chemical reactions is provided (Figure 1C). These chemical reactions are derived in part from the 58 reactions compiled by Hartenfeller *et al.* (24). The comprehensive list of reactions used in ChemoDOTS, along with their SMARTS representations, is available in Supplementary Tables S3–S5. These chemical reactions are categorized into three groups based on their relevance. Users are required to select at least one compatible chemical reaction before proceeding to the next step. In the next step, a summary is provided, including the 2D structure of the initial fragment, key molecular descriptors, the SMILES code, the chemical function for fragment growing, and the selected compatible chemical reactions (Figure 1D).

In the current version, the database contains 501 542 BBs from MolPort (https://www.molport.com/) and 988 112 from Enamine (https://enamine.net/building-blocks). In future releases, we plan to incorporate collections of BBs from other providers such as ChemDiv (https://www.chemdiv.com/catalog/building-blocks), ChemSpace (https://chem-space.com/building-blocks) or Otava (https://www.otavachemicals.com/products/chemical-building-blocks). Additionally, we aim to enable users to upload their own collections of BBs.

All compounds compatible with the selected parameters are then generated within seconds to minutes depending on the number of BBs that are compatible with the chosen reactions (Figure 1D). On average 1000–1500 compounds are generated per second.

### Statistics of the raw library

Upon generating the raw virtual library, a new page is displayed presenting general statistics about the library such as the number of generated compounds and duplicates. For each selected reaction, similar statistics are provided along with its overall contribution to the entire chemical space of the raw library. In addition, a subset of the generated compounds can be displayed in 2D format. At this stage, the raw library and the corresponding BBs can be downloaded in SDF and SMILES formats.

### Postprocessing

**Figure 2.**
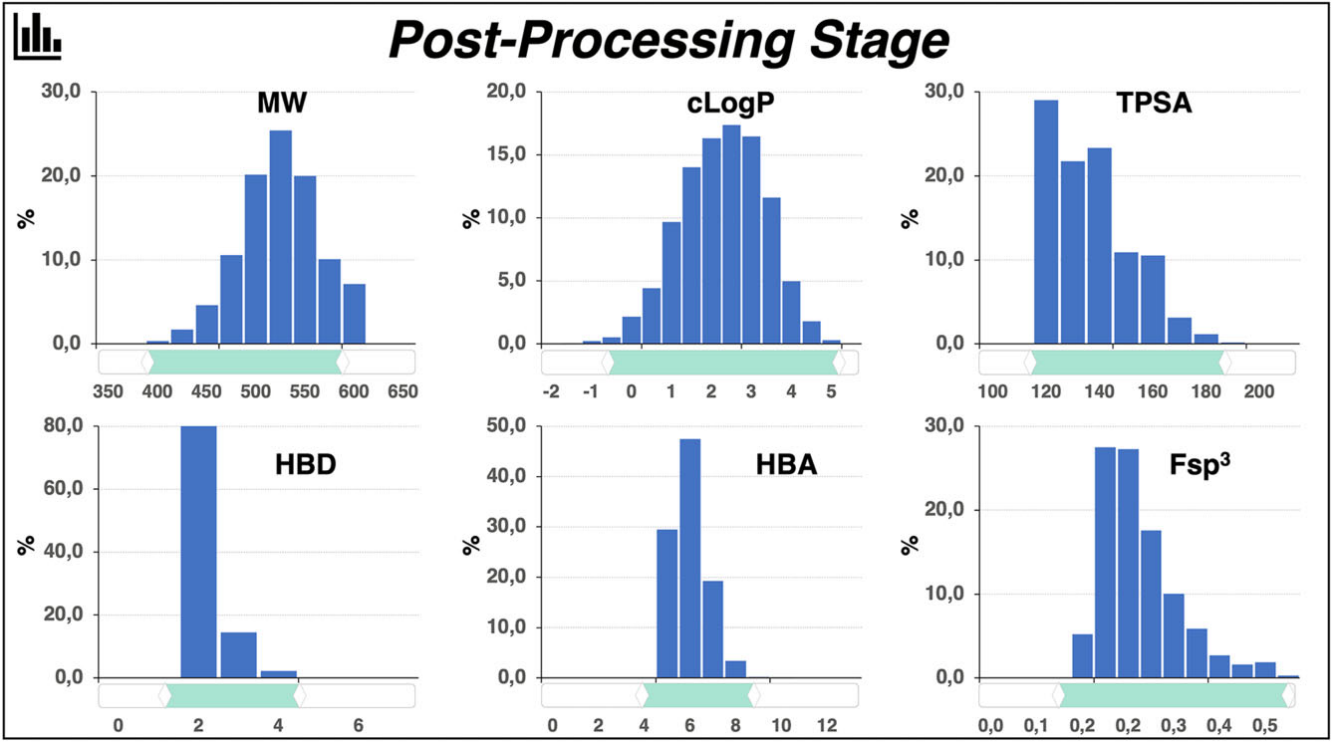
Postprocessing of the raw chemical library. The raw library generated in the initial stage of the process can be refined by adjusting molecular descriptors commonly used in the early phases of drug discovery. These descriptors include molecular weight (MW), logarithm of n-octanol-water partition (cLogP), topological polar surface area (TPSA), number of hydrogen bond donors (HBD), number of hydrogen bond acceptors (HBA), and fraction of sp^3^ carbons (Fsp^3^), an indicator of the three dimensionality of molecules. For each property, the distribution is displayed, and a slider allows users to adjust the minimum and maximum values of each descriptor. The total number of molecules in the filtered library is automatically updated.

The subsequent steps are optional and are highlighted in Figure 2. In the post-processing stages, users have the possibility to fine-tune the drug-like properties of the compounds in the virtual library. The distributions of six key molecular descriptors are available encompassing MW, cLogP, HBD, HBA, TPSA, and Fsp^3^. Additional common descriptors will be added in future release or upon request. Users can easily modify the range of each value by using the graphical sliders provided below each plot. The filtered chemical library can then be downloaded in SDF and SMILES formats.

### Generation of ready-to-dock files

In the last step, major microspecies, atomic types, partial charges, and one 3D-conformer of each compound in the virtual library are computed. The resulting final 3D library can be downloaded in SDF and ready-to-dock mol2 formats.

### Retrospective case study

**Figure 3.**
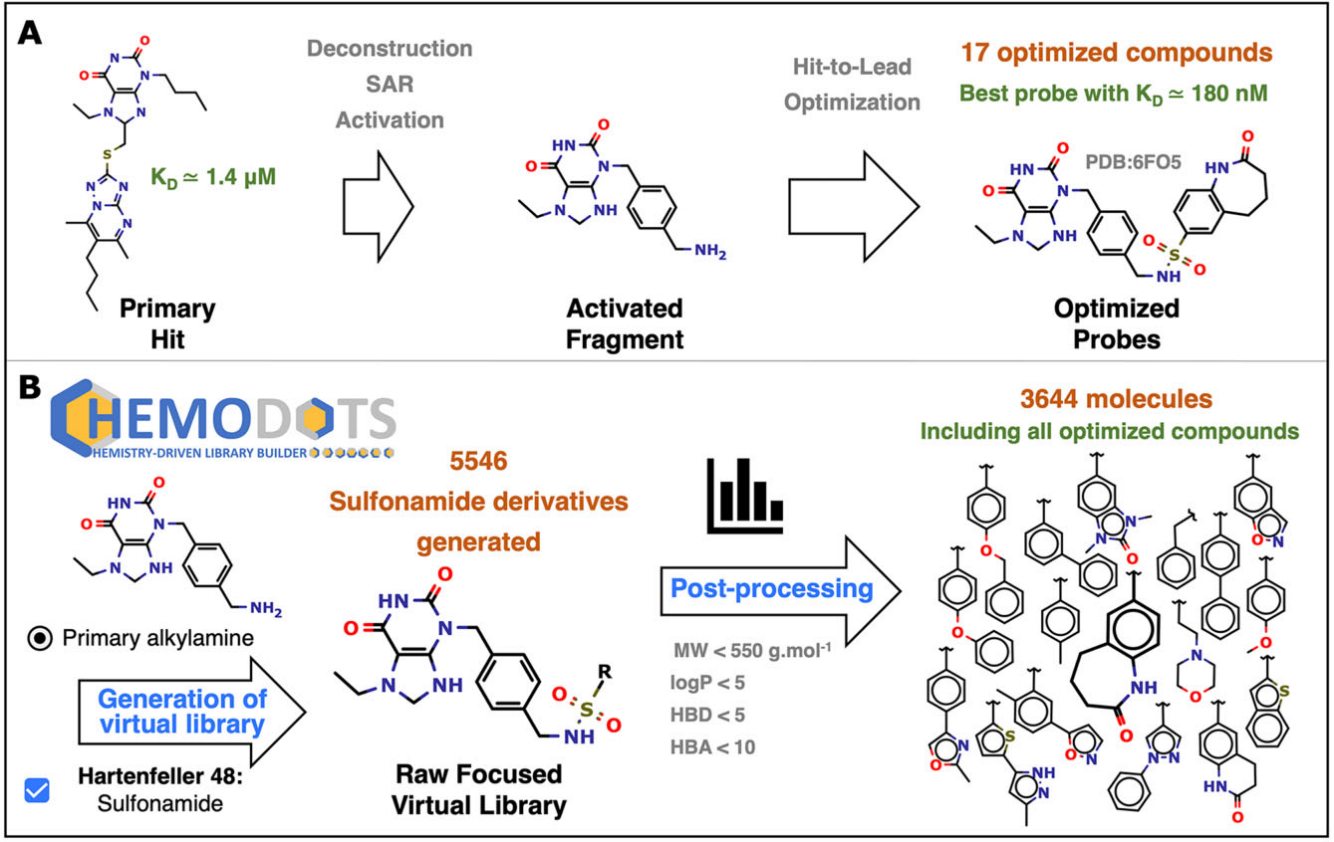
Case study using a retrospective identification of bromodomain inhibitors. (**A**) We previously identified a xanthin derivative inhibitor of bromodomains through high throughput screening (52). This primary hit (K_D_ ≈ 1.4 μM) was deconstructed to find the smallest fragment able to bind the target. This fragment was optimized and activated with a reactive primary amino group (36). Subsequently, this activated fragment was optimized using our *in-house* hit-to-lead approach called DOTS resulting in 17 compounds with improved affinities including a sub-micromolar probe exhibiting almost a 2-log improvement (36,43). (**B**) To test the ability to generate such compounds, the activated fragment was uploaded in SMILES format on the ChemoDOTS server. The primary alkylamine function was automatically detected as the only reactive function on the molecule. The sulfonamide reaction and the MolPort BBs were then selected to generate the raw virtual library resulting in 5546 sulfonamide derivatives. A post-processing filtering was applied to lead to 3644 compounds which include all 17 optimized compounds identified during the hit-to-lead optimization.

We previously engaged in a fragment-based drug design program followed by hit-to-lead optimization to develop chemical probes targeting bromodomain proteins from the BET family (36,43,52). Bromodomains are protein modules that play a crucial role in gene regulation by recognizing and binding to acetylated lysine residues on histone proteins. The development of bromodomain inhibitors is a promising strategy in cancer treatment offering a targeted approach to modulate gene expression and hinder the growth of cancer cells by disrupting crucial molecular interactions involved in the regulation of gene transcription. Briefly, several primary hits were identified through high throughput screening of an in-house chemical library dedicated to protein–protein interactions (52). The most potent inhibitor (K_D_ ≈ 1.4 μM) was deconstructed, and its affinity was improved using structure–activity relationship (SAR) studies (Figure 3A). The resulting fragment was activated with a primary amine to allow growing optimization using our in-house strategy that combines virtual screening with automated real-world synthesis in a platform called Diversity-Oriented Target-focused Synthesis (DOTS, (36)). This study led to the design of 17 compounds with improved affinities. The affinity of the most potent compound was improved more than 60 times compared to the initial fragment and its binding mode was validated by X-ray crystallography (PDB: 6FO5) (36).

We tested the ability of ChemoDOTS to generate these compounds. The initial fragment activated with a reactive primary amine function was uploaded into the Marvin JS sketcher (Figure 3B). As expected, the primary amine function was automatically detected as the only chemical function on the fragment. We then selected the sulfonamidation reaction (#48 from Hartenfeller set of reactions) and the MolPort BBs to generate the raw chemical library. The process resulted in 5546 molecules in 5 s after removal of duplicates. We then applied a standard filtering step using Lipinski-like thresholds (MW < 550 g mol^−1^; log *P* < 5; number of hydrogen bond donors < 5; number of hydrogen bond acceptors < 10) leading to 3644 molecules. All 17 compounds previously identified during the hit-to-lead optimization were present in the final virtual library. All files generated during this retrospective case have been deposited in a zenodo archive (https://dx.doi.org/10.5281/zenodo.10701800).

## Discussion

In summary, ChemoDOTS is a user-friendly web server that enables the generation and exploration of a vast chemical space around an initial hit molecule by combining molecular building blocks and predefined chemistry rules. Over the last few years, the concept of large chemical space exploration has come to the forefront of drug discovery (35,53). All compounds in the raw virtual library should be easily amenable to organic synthesis in one or two steps. This is achieved by using a collection of robust chemical reactions commonly used in the hit-to-lead optimization phase in the pharmaceutical industry. The drug-like properties of the generated compounds can be improved by adjusting various molecular descriptors. The final chemical library is ready for synthesis or further investigation using either ligand-based or structure-based virtual screening experiments. The high level of parallelization ensures the rapid generation of molecules at more than one thousand molecules generated per second. The global architecture of the server as a database allows efficient web server maintenance and scalability. The current version of ChemoDOTS relies on a collection of more than 500 000 BBs from MolPort and almost a million from Enamine. However, the virtual chemical space covered by ChemoDOTS can be easily extended through the integration of additional BBs from other providers or user-defined sources. Additionally, the incorporation of new chemical reactions including those used in click chemistry offers further avenues for expansion (54,55). The post-processing stage allows users to custom-filter the final collection of compounds by physico-chemical properties in line with the requirements of their drug discovery project. New molecular descriptors can be easily integrated at this stage in future releases of the server. We also anticipate benefiting from users’ feedback to upgrade ChemoDOTS and to deliver a better service. To our knowledge, ChemoDOTS is the only freely accessible functional and maintained web server to combine the design of medchem-compatible focused virtual libraries with an integrated graphical postprocessing analysis. Therefore, it creates a valuable resource for scientists engaged in H2L optimization, particularly for those who may lack the chemoinformatics knowledge required for tasks such as library enumeration, filtering and preparation for subsequent docking experiments.

## Supplementary Material

gkae326_Supplemental_File

## Data Availability

The ChemoDOTS web server is freely available at https://chemodots.marseille.inserm.fr/. All scripts used in ChemoDOTS backend have been deposited on GitHub (https://github.com/iSCBTeam/ChemoDOTS). Permanent DOI of the code used for ChemoDOTS: Backend: https://doi.org/10.6084/m9.figshare.25585089 Frontend: https://doi.org/10.6084/m9.figshare.25585092 The list of chemical functions, the list of reactions, and the final BBs from MolPort and Enamine (in SMILES format) are available via Zenodo repository at https://dx.doi.org/10.5281/zenodo.10776787. Files from the retrospective test case can be accessed through the Zenodo repository at https://dx.doi.org/10.5281/zenodo.10701800.

## Notes

Present address: Laurent Hoffer, Drug Discovery Program, Ontario Institute for Cancer Research, Toronto, Ontario M5G 0A3, Canada.
