# Supplementary material for: ChemoDOTS: a web server to design chemistry-driven focused libraries
Source: Nucleic Acids Res. 2024 Apr 30;52(W1):W461–8. doi: 10.1093/nar/gkae326 (PMC11223810; doi:10.1093/nar/gkae326)
Supplement: gkae326_Supplemental_File [file gkae326_supplemental_file.pdf]

ChemoDOTS: A Web Server to Design Chemistry-Driven Focused Libraries

Supplementary Information

AUTHORS

Laurent Hoffer[1,2†], Guillaume Charifi-Hoareau[1†], Sarah Barelier[1], Stéphane Betzi[1], Thomas Miller[1], Xavier Morelli[1,*] and Philippe Roche[1,*]

[1] CRCM, CNRS, Inserm, Institut Paoli-Calmettes, Aix-Marseille Univ, Marseille, 13273, France;

* To whom correspondence should be addressed. Tel: +33 (0)486 977 335; Fax: +33 (0)486 977 499; Email: philippe.roche@inserm.fr

Correspondence may also be addressed to Dr Xavier Morelli, Email: xavier.morelli@inserm.fr

[2] Present Address: Laurent Hoffer, Drug Discovery Program, Ontario Institute for Cancer Research, Toronto, Ontario, M5G 0A3, Canada

† Joint Authors

Content

Table S1: List of alkyl chemical functions recognized by ChemoDOTS and associated SMILES.

Table S2: List of aryl chemical functions recognized by ChemoDOTS and associated SMILES.

Table S3: List of chemical reactions implemented in ChemoDOTS and associated SMARTS (rules 1 to 28).

Table S4: List of chemical reactions implemented in ChemoDOTS and associated SMARTS (rules 29 to 58).

Table S5: List of *in house* chemical reactions implemented in ChemoDOTS and associated SMARTS (rules 61 to 70).

Figure S1: 2D Structures of chemical functions automatically detected by ChemDOTS.

Table S1: List of alkyl chemical functions recognized by ChemoDOTS and associated SMILES. The list has also been deposited to Zenodo (https://zenodo.org/records/10776787).

Function	SMILE	Function	SMILE
alkyl_boronate	OB(O)C	alpha-haloketone	CC(=O)CBr
primary_alkyl_amine	CN	beta-haloketone	CC(=O)CCBr
secondary_alkyl_amine	CNC	alkyl_epoxyde	C1OC1C
tertiary_alkyl_amine	CN(C)C	alkyl_acylchloride	CC(Cl)=O
alkyl_nitrile	CC#N	alkyl_thioether	CSC
N-alkyl_aziridine	C1NC1C	alkyl_thiol	CS
N-alkyl_imine	C(=N)C	alkyl_amide	CC(N)=O
N-alkyl_azide	[N-]=[N+]=NC	alkyl_isocyanate	CN=C=O
N-alkyl_amidine	CC(N)=N	alkyl_nitro	C[N+]([O-])=O
alkyl_hydrazine	CNN	alkyl_imide	CC(=O)NC(=O)C
alkyl_alcohol	CO	alkyl_thioester	CC(=S)OC
alkyl_carboxylic_acid	CC(O)=O	alkyl_vinylsulfonyl	CS(=O)(=O)C=C
alkyl_aldehyde	CC(=O)	alkyl_ester_sulfonate	CS(=O)(=O)OC
alkyl_ketone	CC(=O)C	alkyl_sulfonylhalide	CS(Cl)(=O)=O
alkyl_ester	CC(=O)OC	alkyl_thioamide	CC(N)=S
alkyl_ether	COC	alkyl_isothiocyanate	CN=C=S
alkyl_Michael_acceptor	CC(=O)C=C	alkyl_sulfonamide	CS(N)(=O)=O
alkyl_anhydride	CC(=O)OC(=O)C	alkyl_alkyne	CC#C
alkyl_1,3-dicarbonyl	CC(=O)CC(=O)C	alkyl_alkene	CC=C
alkyl_1,4-dicarbonyl	CC(=O)CCC(=O)C	alkyl_halide_Cl	CCl
		alkyl_halide_Br	CBr

Table S2: List of aryl chemical functions recognized by ChemoDOTS and associated SMILES. The list has also been deposited to Zenodo (https://zenodo.org/records/10776787).

Function	SMILE	Function	SMILE
aryl_boronate	OB(O)c1ccccc1	aryl_epoxyde	C1OC1c1ccccc1
primary_aryl_amine	c1ccccc1N	aryl_acylchloride	c1ccccc1C(Cl)=O
secondary_aryl_amine	c1ccccc1NC	aryl_thioether	CSc1ccccc1
tertiary_aryl_amine	c1ccccc1N(C)C	aryl_thiol	c1ccccc1S
aryl_nitrile	c1ccccc1C#N	aryl_amide	c1ccccc1C(N)=O
N-aryl_aziridine	C1NC1c1ccccc1	aryl_isocyanate	c1ccccc1N=C=O
N-aryl_imine	C(=N)c1ccccc1	aryl_nitro	c1ccccc1[N+]([O-])=O
N-aryl_azide	[N-]=[N+]=Nc1ccccc1	aryl_imide	c1ccccc1C(=O)NC(=O)c1ccccc1
N-aryl_amidine	c1ccccc1C(N)=N	aryl_thioester	c1ccccc1C(=S)OC
aryl_hydrazine	c1ccccc1NN	aryl_vinylsulfonyl	c1ccccc1S(=O)(=O)C=C
aryl_alcohol	c1ccccc1O	aryl_ester_sulfonate	c1ccccc1S(=O)(=O)Oc1ccccc1
aryl_carboxylic_acid	c1ccccc1C(O)=O	aryl_sulfonylhalide	c1ccccc1S(Cl)(=O)=O
aryl_aldehyde	c1ccccc1C(=O)	aryl_isothiocyanate	c1ccccc1N=C=S
aryl_ketone	c1ccccc1C(=O)c1ccccc1	aryl_sulfonamide	c1ccccc1S(N)(=O)=O
aryl_ester	c1ccccc1C(=O)OC	aryl_alkyne	c1ccccc1C#C
aryl_ether	COc1ccccc1	aryl_alkene	c1ccccc1C=C
aryl_Michael_acceptor	c1ccccc1C(=O)C=C	aryl_halide_Cl	c1ccccc1Cl
aryl_anhydride	c1ccccc1C(=O)OC(=O)c1ccccc1	halo_pyrimidine_Cl	Clc1ncccn1
aryl_1,3-dicarbonyl	c1ccccc1C(=O)CC(=O)c	aryl_halide_Br	c1ccccc1Br
aryl_1,4-dicarbonyl	c1ccccc1C(=O)CCC(=O)c	halo_pyrimidine_Br	Brc1ncccn1

Table S3: List of chemical reactions (rules 1 to 28 from Hartenfeller *et al.,*), implemented in ChemoDOTS. The SMARTS definition and name of each reaction is provided.

The list has been deposited to Zenodo (https://zenodo.org/records/10776787).

Rule	SMARTS	Reaction
1	[cH1:1]1:[c:2](-[CH2:7]-[CH2:8]-[NH2:9]):[c:3]:[c:4]:[c:5]:[c:6]:1.[#6:11]-[CH1;R0:10]=[OD1]>>[c:1]12:[c:2](-[CH2:7]-[CH2:8]-[NH1:9]-[C:10]-2(-[#6:11])):[c:3]:[c:4]:[c:5]:[c:6]:1	Pictet-Spengler
2	[c;r6:1](-[NH1;$(N-[#6]):2]):[c;r6:3](-[NH2:4]).[#6:6]-[C;R0:5](=[OD1])-[#8;H1,$(O-[CH3])]>>[c:3]2:[c:1]:[n:2]:[c:5](-[#6:6]):[n:4]@2	Benzimidazole derivatives carboxylic-acid/ester
3	[c;r6:1](-[NH1;$(N-[#6]):2]):[c;r6:3](-[NH2:4]).[#6:6]-[CH1;R0:5](=[OD1])>>[c:3]2:[c:1]:[n:2]:[c:5](-[#6:6]):[n:4]@2	Benzimidazole derivatives aldehyde
4	[c;r6:1](-[SH1:2]):[c;r6:3](-[NH2:4]).[#6:6]-[CH1;R0:5](=[OD1])>>[c:3]2:[c:1]:[s:2]:[c:5](-[#6:6]):[n:4]@2	Benzothiazole
5	[c:1](-[OH1;$(Oc1ccccc1):2]):[c;r6:3](-[NH2:4]).[c:6]-[CH1;R0:5](=[OD1])>>[c:3]2:[c:1]:[o:2]:[c:5](-[c:6]):[n:4]@2	Benzoxazole arom-aldehyde
6	[c;r6:1](-[OH1:2]):[c;r6:3](-[NH2:4]).[#6:6]-[C;R0:5](=[OD1])-[OH1]>>[c:3]2:[c:1]:[o:2]:[c:5](-[#6:6]):[n:4]@2	Benzoxazole carboxylic-acid
7	[#6:6]-[C;R0:1](=[OD1])-[CH1;R0:5](-[#6:7])-[*;#17,#35,#53].[NH2:2]-[C:3]=[SD1:4]>>[c:1]2(-[#6:6]):[n:2]:[c:3]:[s:4][c:5]([#6:7]):2	Thiazole
8	[c:1](-[C;$(C-c1ccccc1):2](=[OD1:3])-[OH1]):[c:4](-[NH2:5]).[N;!H0;!$(N-N);!$(N-C=N);!$(N(-C=O)-C=O):6]-[C;H1,$(C-[#6]):7]=[OD1]>>[c:4]2:[c:1]-[C:2](=[O:3])-[N:6]-[C:7]=[N:5]-2	Niementowski quinazoline
10	[CH0;$(C-[#6]):1]#[NH0:2].[C;A;!$(C=O):3]-[*;#17,#35,#53]>>[C:1]1=[N:2]-N(-[C:3])-N=N-1	Tetrazole connect regioisomere 1
11	[CH0;$(C-[#6]):1]#[NH0:2].[C;A;!$(C=O):3]-[*;#17,#35,#53]>>[C:1]1=[N:2]-N=N-N-1(-[C:3])	Tetrazole connect regioisomere 2
12	[CH0;$(C-[#6]):1]#[CH1:2].[C;H1,H2;A;!$(C=O):3]-[*;#17,#35,#53,OH1]>>[C:1]1=[C:2]-N(-[C:3])-N=N-1	Huisgen Cu-catalyzed 1,4-subst
13	[CH0;$(C-[#6]):1]#[CH1:2].[C;H1,H2;A;!$(C=O):3]-[*;#17,#35,#53,OH1]>>[C:1]1=[C:2]-N=NN(-[C:3])-1	Huisgen Ru-catalyzed 1,5 subst
14	[CH0;$(C-[#6]):1]#[CH0;$(C-[#6]):2].[C;H1,H2;A;!$(C=O):3]-[*;#17,#35,#53,OH1]>>[C:1]1=[C:2]-N=NN(-[C:3])-1	Huisgen disubst-alkyne
15	[CH0;$(C-[#6]):1]#[NH0:2].[NH2:3]-[NH1:4]-[CH0;$(C-[#6]);R0:5]=[OD1]>>[N:2]1-[C:1]=[N:3]-[N:4]-[C:5]=1	1,2,4-triazole acetohydrazide
16	[CH0;$(C-[#6]):1]#[NH0:2].[CH0;$(C-[#6]);R0:5](=[OD1])-[#8;H1,$(O-[CH3]),$(O-[CH2]-[CH3])]>>[N:2]1-[C:1]=N-N-[C:5]=1	1,2,4-triazole carboxylic-acid/ester
18	[c:1](-[C;$(C-c1ccccc1):2](=[OD1:3])-[CH3:4]):[c:5](-[OH1:6]).[C;$(C1-[CH2]-[CH2]-[N,C]-[CH2]-[CH2]-1):7](=[OD1])>>[O:6]1-[c:5]:[c:1]-[C:2](=[OD1:3])-[C:4]-[C:7]-1	Spiro-chromanone
19	[#6;!$([#6](-C=O)-C=O):4]-[CH0:1](=[OD1])-[C;H1&!$(C-[*;!#6])&!$(C-C(=O)O),H2:2]-[CH0;R0:3](=[OD1])-[#6;!$([#6](-C=O)-C=O):5].[NH2:6]-[N;!H0;$(N-[#6]),H2:7]>>[C:1]1(-[#6:4])-[C:2]=[C:3](-[#6:5])-[N:7]-[N:6]=1	Pyrazole
20	[c;r6:1](-[C;$(C=O):6]-[OH1]):[c;r6:2]-[C;H1,$(C-C):3]=[OD1].[NH2:4]-[NH1;$(N-[#6]);!$(NC=[O,S,N]):5]>>[c:1]1:[c:2]-[C:3]=[N:4]-[N:5]-[C:6]-1	Phthalazinone
21	[#6:5]-[C;R0:1](=[OD1])-[C;H1,H2:2]-[C;H1,H2:3]-[C:4](=[OD1])-[#6:6].[NH2;$(N-[C,N]);!$(NC=[O,S,N]);!$(N([#6])[#6]);!$(N~N~N):7]>>[C:1]1(-[#6:5])=[C:2]-[C:3]=[C:4](-[#6:6])-[N:7]-1	Paal-Knorr pyrrole
22	[C;$(C-c1ccccc1):1](=[OD1])-[C;D3;$(C-c1ccccc1):2]~[O;D1,H1].[CH1;$(C-c):3]=[OD1]>>[C:1]1-N=[C:3]-[NH1]-[C:2]=1	Triaryl-imidazole
23	[NH1;$(N-c1ccccc1):1](-[NH2])-[c:5]:[cH1:4].[C;$(C([#6])[#6]):2](=[OD1])-[CH2;$(C([#6])[#6]);!$(C(C=O)C=O):3]>>[C:5]1-[N:1]-[C:2]=[C:3]-[C:4]:1	Fischer indole
24	[NH2;$(N-c1ccccc1):1]-[c:2]:[c:3]-[CH1:4]=[OD1].[C;$(C([#6])[#6]):6](=[OD1])-[CH2;$(C([#6])[#6]);!$(C(C=O)C=O):5]>>[N:1]1-[c:2]:[c:3]-[C:4]=[C:5]-[C:6]:1	Friedlaender chinoline
25	[*;Br,I;$(*c1ccccc1)]-[c:1]:[c:2]-[OH1:3].[CH1:5]#[C;$(C-[#6]):4]>>[c:1]1:[c:2]-[O:3]-[C:4]=[C:5]-1	Benzofuran
26	[*;Br,I;$(*c1ccccc1)]-[c:1]:[c:2]-[SD2:3]-[CH3].[CH1:5]#[C;$(C-[#6]):4]>>[c:1]1:[c:2]-[S:3]-[C:4]=[C:5]-1	Benzothiophene
27	[*;Br,I;$(*c1ccccc1)]-[c:1]:[c:2]-[NH2:3].[CH1:5]#[C;$(C-[#6]):4]>>[c:1]1:[c:2]-[N:3]-[C:4]=[C:5]-1	Indole
28	[#6:6][C:5]#[#7;D1:4].[#6:1][C:2](=[OD1:3])[OH1]>>[#6:6][c:5]1[n:4][o:3][c:2]([#6:1])n1	Oxadiazole

Table S4 : List of chemical reactions (rules 29 to 58 from by Hartenfeller et al.,), implemented in ChemoDOTS. The SMARTS definition and name of each reaction is provided.

The list has been deposited to Zenodo (https://zenodo.org/records/10776787).

Rule	SMARTS	Reaction
29	[#6;$([#6]~[#6]);!$([#6]=O):2][#8;H1:3].[Cl,Br,I]<br>[#6;H2;$([#6]~[#6]):4]>>[CH2:4][O:3][#6:2]	Pictet-Spengler
30	[#6:4]-[C;H1,$([CH0](-[#6])[#6]):1]=[OD1].[N;H2,$([NH1;D2](C)C)<br>;!$(N-[#6]=[*]):3]-[C:5]>>[#6:4][C:1]-[N:3]-[C:5]	Benzimidazole derivatives<br>carboxylic-acid/ester
31	[#6;H0;D3;$([#6](~[#6])~[#6]):1]B(O)O.[#6;H0;D3;$([#6](~[#6])<br>~[#6]):2][Cl,Br,I]>>[#6:2][#6:1]	Benzimidazole derivatives aldehyde
32	[c;H1:3]1:[c:4]:[c:5]:[c;H1:6]:[c:7]2:[nH:8]:[c:9]:[c;H1:1]:[c:2]:1:2.O=[C:10]<br>1[#6;H2:11][#6;H2:12][N:13][#6;H2:14][#6;H2:15]1>><br>[#6;H2:12]3[#6;H1:11]=[C:10]([c:1]1:[c:9]:[n:8]:[c:7]2:[c:6]:<br>[c:5]:[c:4]:[c:3]:[c:2]:1:2)[#6;H2:15][#6;H2:14][N:13]3	Benzothiazole
33	[#6;$([#6]~[#6]);!$([#6]~[S,N,O,P]):1][Cl,Br,I].[Cl,Br,I]<br>[#6;$([#6]~[#6]);!$([#6]~[S,N,O,P]):2]>>[#6:2][#6:1]	Benzoxazole aromatic aldehyde
34	[C;H1&$(C([#6])[#6]),H2&$(C[#6]):1][OH1].<br>[NH1;$(N(C=O)C=O):2]>>[C:1][N:2]	Benzoxazole carboxylic-acid
35	[C;H1&$(C([#6])[#6]),H2&$(C[#6]):1]<br>[OH1].[OH1;$(Oc1ccccc1):2]>>[C:1][O:2]	Thiazole
36	[C;H1&$(C([#6])[#6]),H2&$(C[#6]):1]<br>[OH1].[NH1;$(N([#6])S(=O)=O):2]>>[C:1][N:2]	Niementowski quinazoline
41	[#6;c,$(C(=O)O),$(C#N):3][#6;H1:2]=[#6;H2:1].<br>[#6;$([#6]=[#6]),$(c:c):4][Cl,Br,I]>>[#6:4]/[#6:1]=[#6:2]/[#6:3]	Tetrazole connect regioisomer 1
42	[#6;c,$(C(=O)O),$(C#N):3][#6:2]([#6:5])=[#6;H1;$([#6][#6]):1].<br>[#6;$([#6]=[#6]),$(c:c):4][Cl,Br,I]>>[#6:4][#6;H0:1]=[#6:2]([#6:5])[#6:3]	Tetrazole connect regioisomer 2
43	[#6;$(C=C-[#6]),$(c:c):1][Br,I].[Cl,Br,I][c:2]>>[c:2][#6:1]	Huisgen Cu-catalyzed 1,4-subst
44	[#6:1][C:2]#[#7;D1].[Cl,Br,I][#6;$([#6]~[#6]);!$([#6]([Cl,Br,I])<br>[Cl,Br,I]);!$([#6]=O):3]>>[#6:1][C:2](=O)[#6:3]	Huisgen Ru-catalyzed 1,5- subst
45	[#6:1][C;H1,$([C]([#6])[#6]):2]=[OD1:3].[Cl,Br,I][#6;$([#6]~[#6]);!$([#6]<br>([Cl,Br,I])[Cl,Br,I]);!$([#6]=O):4]>>[C:1][#6:2]([OH1:3])[#6:4]	Huisgen disubst-alkyne
46	[#6;$(C=C-[#6]),$(c:c):1][Br,I].[CH1;$(C#CC):2]>>[#6:1][C:2]	1,2,4-triazole acetohydrazide
47	[C;$(C=O):1][OH1].[N;$(N[#6]);!$(N=*);!$([N-]);!$(N#*);!$([ND3]);<br>!$([ND4]);!$(N[O,N]);!$(N[C,S]=[S,O,N]):2]>>[C:1][N+0:2]	1,2,4-triazole carboxylic-acid/ester
48	[S;$(S(=O)(=O)[#6,N]):1][Cl,OH,O-].[N;$(N[#6]);!$(N=*);!$([N-]);!$(N#*);<br>!$([ND3]);!$([ND4]);!$(NO);!$(N[C,S]=[S,O,N]):2]>>[S:1][N+0:2]	Spiro-chromanone
49	[c:1]B(O)O.[nH1;+0;r5;!$(n[#6]=[O,S,N]);!$(n~n~n);<br>!$(n~n~c~n);!$(n~c~n~n):2]>>[c:1][n:2]	Pyrazole
50	[#6:3]-[C;H1,$([CH0](-[#6])[#6]);!$(CC=O):1]=[OD1].[Cl,Br,I][C;H2;<br>$(C-[#6]);!$(CC[I,Br]);!$(CCO[CH3]):2]>>[C:3][C:1]=[C:2]	Phthalazinone
51	[Cl,Br,I][c;$(c1:[c,n]:[c,n]:[c,n]:[c,n]:[c,n]:1):1].[N;$(NC)&!$(N=*)&<br>!$([N-])&!$(N#*)&!$([ND3])&!$([ND4])<br>&!$(N[c,O])&!$(N[C,S]=[S,O,N]),H2&$(Nc1:[c,n]:[c,n]:[c,n]:[c,n]:[c,n]:1):2]>>[c:1][N:2]	Paal-Knorr pyrrole
52	[C;$(C([#6])[#6;!$([#6]Br)]):4](=[OD1])[CH;$(C([#6])[#6]):5]Br.[#7;H2:3][C;<br>$(C(=N)(N)[c,#7]):2]=[#7;H1;D1:1]>>[C:4]1=[CH0:5][NH:3][C:2]=[N:1]1	Triaryl-imidazole
53	[c;$(c1[c;$(c[C,S,N](=[OD1])[*;R0;!OH1])]cccc1):1][C;<br>$(C(=O)[O;H1])].[c;$(c1aaccc1):2][Cl,Br,I]>>[c:1][c:2]	Fischer indole
54	[c;!$(c1ccccc1);$(c1[n,c]c[n,c]c[n,c]1):1][Cl,F].[N;$(NC);!$(N=*);!$([N-]);<br>!$(N#*);!$([ND3]);!$([ND4]);!$(N[c,O]);!$(N[C,S]=[S,O,N]):2]>>[c:1][N:2]	Friedlaender chinolin
55	[c;$(c1c(N(~O)~O)cccc1):1][Cl,F].[N;$(NC);!$(N=*);!$([N-]);!$(N#*);<br>!$([ND3]);!$([ND4]);!$(N[c,O]);!$(N[C,S]=[S,O,N]):2]>>[c:1][N:2]	Benzofuran
56	[c;$(c1ccc(N(~O)~O)cc1):1][Cl,F].[N;$(NC);!$(N=*);!$([N-]);!$(N#*);<br>!$([ND3]);!$([ND4]);!$(N[c,O]);!$(N[C,S]=[S,O,N]):2]>>[c:1][N:2]	Benzothiophene
57	[N;$(N-[#6]):3]=[C;$(C=O):1].[N;$(N[#6]);!$(N=*);!$([N-]);!$(N#*);!$([ND3]);<br>!$([ND4]);!$(N[O,N]);!$(N[C,S]=[S,O,N]):2]>>[N:3]-[C:1]-[N+0:2]	Indole
58	[N;$(N-[#6]):3]=[C;$(C=S):1].[N;$(N[#6]);!$(N=*);!$([N-]);!$(N#*);!$([ND3]);<br>!$([ND4]);!$(N[O,N]);!$(N[C,S]=[S,O,N]):2]>>[N:3]-[C:1]-[N+0:2]	Oxadiazole

Table S5 : List of in house chemical reactions implemented in ChemoDOTS. The SMARTS definition and name of each reaction is provided.

The list has also been deposited to Zenodo (https://zenodo.org/records/10776787).

Rule	SMARTS	Reaction
61	[c;r6:1](-[NH2:2]):[c;r6:3](-[NH2:4]).[#6:6]-[CH1;R0:5](=[OD1])>>[c:3]2:[c:1]:[nH:2]:[c:5](-[#6:6]):[n:4]@2	Benzimidazole aldehyde (fuzzy)
62	[c;r6:1](-[NH2:2]):[c;r6:3](-[NH2:4]).[#6:6]-[C;R0:5](=[OD1])-[#8;H1,$(O-[CH3])]>>[c:3]2:[c:1]:[nH:2]:[c:5](-[#6:6]):[n:4]@2	Benzimidazole carboxylic-acid-ester (fuzzy)
63	[CH2;$([#6]~[#6]):1][Br,I].[#8;H1:2][#6;$([#6]~[#6]);!$([#6]=O):3]>>[C:1][O:2][#6:3]	Williamson-like alcohol
64	[CH2;$([#6]~[#6]):1][Br,I].[#16;H1:2][#6;$([#6]~[#6]);!$([#6]=O):3]>>[C:1][S:2][#6:3]	Williamson-like thiol
65	[CH2;$([#6]~[#6]):1][Br,I].[#7;H2:2][#6;$([#6]~[#6]);!$([#6]=O):3]>>[C:1][N:2][#6:3]	Williamson-like amine
66	[c:1][Sn](C)(C)(C).[Cl,Br,I][c:2]>>[c:1][c:2]	Stille organo-stannane
67	[S;$(S(=O)(=O)[#6]):1][Cl,Br].[OH;$(O[#6]);!$(OC=O):2]>>[S:1][O:2]	Sulfonyl ester
68	[C;$(C=O):1][Cl,Br].[N;$(N[#6]);!$(N=*);!$([N-]);!$(N#*);!$([ND3]);!$([ND4]);!$(N[O,N]);!$(N[C,S]=[S,O,N]):2]>>[C:1][N+0:2]	Amide acyl-chloride
69	[c:3][#6;H1:2]=[#6;H2:1].[#6;$([#6]=[#6]),$(c:c):4][Cl,Br,I]>>[#6:4]/[#6:1]=[#6:2]/[#6:3]	Heck terminal vinyl (fuzzy)
70	[C;$(C=O):1][OH1].[OH1;$(O[#6]);!$(OC=O):2]>>[C:1][O:2]	Ester

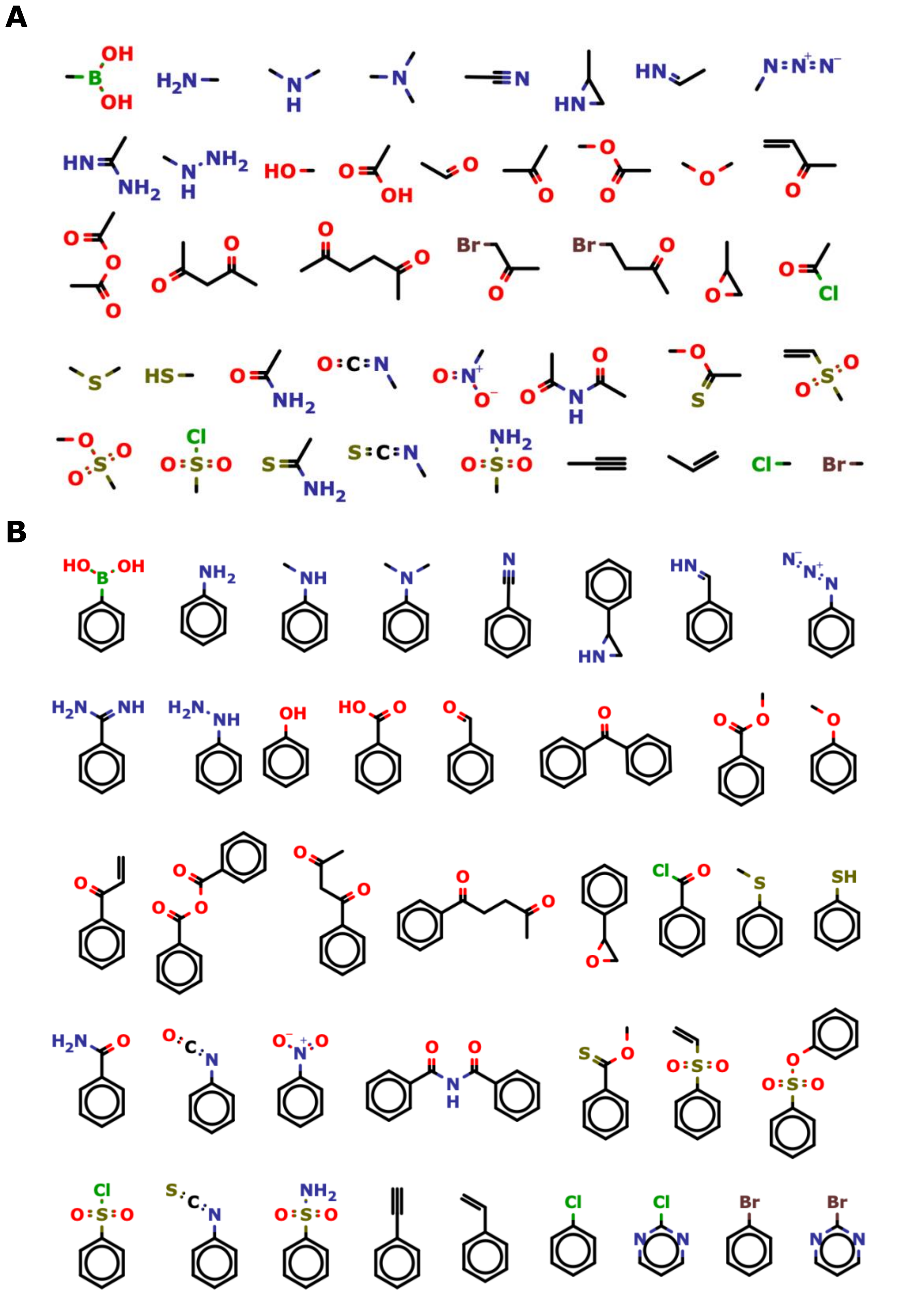

Figure S1: 2D Structures of chemical functions automatically detected by ChemDOTS. A: alkyl functions. **B:** Aryl functions. The complete list of chemical functions with their corresponding smiles is shown in Tables S1 and S2.
